# Hydraulic properties of bentonite buffer material beyond 100 °C

**DOI:** 10.1016/j.heliyon.2023.e18447

**Published:** 2023-07-22

**Authors:** Seok Yoon, Jun-Seo Jeon, Gi-Jun Lee

**Affiliations:** a Disposal Safety Evaluation Research Division, Korea Atomic Energy Research Institute (KAERI), Daejeon, 34057, Republic of Korea; b Department of Geotechnical Engineering Research, Korea Institute of Civil Engineering and Building Technology (KICT), Goyang, 10223, Republic of Korea

**Keywords:** Bentonite buffer material, Hydraulic conductivity, Soil–water characteristic curve, Suction

## Abstract

Bentonite buffer materials are important components of engineered barrier systems for the disposal of high-level radioactive waste produced during nuclear power generation. The design temperature of the buffer material is < 100 °C, and increasing the design temperature can reduce the required disposal area. This characteristic necessitates the evaluation of the thermal–hydraulic–mechanical properties of the buffer at temperatures above 100 °C to increase its target temperature. Therefore, the hydraulic properties of Gyeongju (KJ) bentonite buffer material were evaluated in this study, including the soil–water characteristic curve (SWCC) and hydraulic conductivity. An experimental system was manufactured to measure the suction and saturated hydraulic conductivity of KJ bentonite buffer material above 100 °C; the relative humidity of KJ bentonite buffer material was measured at 25–149 °C with an initial water content of 0, 0.06, and 0.12 under constant saturation conditions. The suction decreased as the temperature increased (10%–25% reduction at 99 °C-149 °C). The Van-Genuchten SWCC fitting parameters were also derived at 25 °C-149 °C using previously reported and newly generated experimental results, and the applicability of the modified Van-Genuchten SWCC model in this temperature range was verified. The hydraulic conductivity was proportional to temperature up to 100 °C, in agreement with the theoretical model results. Between 100 °C and 150 °C, the hydraulic conductivity increased nonlinearly because of molecular motion and structural changes inside the sample.

## Nomenclature

**Greek letters**
*ψ*: water potential
*ψ*_*t*_: total water potential
*ψ*_*p*_: pressure potential
*ψ*_*o*_: osmotic pressure
*ψ*_*m*_: matric pressure
*ψ*_*g*_: gravimetric potential
*s*: suction (MPa)
*RH*: relative humidity (%)
*R*: gas constant (J/(mol·K))
$\nu_{\omega 0}$: specific volume of water (m^3^/kg)
$\omega_{\nu }$: molar mass of water (kg/Kmol)
*T*_*a*_: absolute temperature (K)
*T*: temperature (°C)
*K*: hydraulic conductivity (m/s)
*Q*: flow rate (m^3^/s)
*L*: thickness of the sample (m)
*Δh*: hydraulic head difference (MPa)
*A*: cross-sectional area (m^2^)
*ω*: water content (%)
*n*: porosity
*a*: fitting parameter
*b*: fitting parameter
*c*: fitting parameter
*α*: fitting parameter
*λ*: fitting parameter
*k*_*t*_: hydraulic conductivity according to temperature (m/s)
*ν*: kinematic viscosity

## 1 Introduction

The required site area of high-level radioactive waste (HLW) disposal repositories is mainly dependent on the limit temperature of the bentonite buffer material. Currently, most countries operate using an upper limit temperature of 100 °C [[1], [2], [3], [4]], which when exceeded, deteriorates the constituent minerals of bentonite and diminishes its functional properties. Nevertheless, raising this limit temperature beyond 100 °C would drastically reduce the area required for the disposal of HLW.

Thus, several studies are actively investigating the deterioration of bentonite at temperatures above 100 °C and the consequential changes in its thermal–hydraulic–mechanical–chemical properties [[5], [6], [7], [8]]. The hydraulic properties of the bentonite buffer material are mainly represented by hydraulic conductivity and soil–water characteristic curves (SWCCs). Hydraulic conductivity refers to the rate at which groundwater seeps through the pores of a buffer material [9]. It is a measure of whether nuclide movement is mainly dependent on diffusion [10]. An SWCC represents the saturated and unsaturated behaviors of a bentonite buffer material by measuring its suction ability according to its water content [10]. However, the influence of temperature on the suction and hydraulic conductivity of bentonite buffer material has not been thoroughly investigated beyond 100 °C [[10], [11], [12], [13]]. Particularly, in Korea, research on bentonite buffer materials have focused on Ca-type KJ bentonite (produced in the Gyeongju region of Korea) [10,14,15]. The geotechnical and mineralogical properties of KJ bentonite are different from those of Na-type bentonite. Moreover, the hydraulic properties of KJ bentonite at temperatures beyond 100 °C remain uninvestigated, which is necessary to evaluate its applicability as a buffer material.

This study aimed to investigate the hydraulic properties of KJ bentonite buffer materials with varying water contents over a temperature range of 20 °C-150 °C to evaluate their applicability as buffer materials for HLW disposal repositories based on the hydraulic properties obtained from laboratory experiments. The suction of the KJ bentonite buffer material was measured experimentally according to the water content and temperature variation. The SWCC data reported by Yoon et al. [14], which consider temperatures up to 100 °C, were used to construct an SWCC model appropriate for the temperature range used in the current study. Additionally, the hydraulic conductivity of KJ bentonite buffer material was experimentally measured at 20 °C-150 °C to validate the model. Finally, the hydraulic properties of the KJ bentonites obtained through this study were compared with an Na-type bentonite (MX-80).

## 2 Experimental methods

### 2.1 Materials

The KJ bentonite was produced by Clariant (Gyeongju, Korea) and comprised 60.5%–63.4% montmorillonite, 19.4–22.8% feldspar, and other minerals such as quartz, cristobalite, calcite, and heulandite, as shown in Table 1 [16]. Based on the unified soil classification system, the KJ bentonite can be classified as a high-plasticity clay. The chemical composition of KJ bentonite is as follows: SiO_2_, Al_2_O_3_, and 5–6 times more CaO than NaO.

**Table 1:** Quantitative XRD analysis of KJ bentonite [ [16]].

Bentonite	KJ bentonite powders (%)			
Sample no.	1	2	3	Avg.
Montmorillonite	63.4	61.7	60.5	61.9
Albite	19.4	22.8	20.4	20.9
Quartz	5.8	4.9	5.3	5.3
Cristobalite	4.0	4.5	3.7	4.1
Calcite	4.3	3.3	6.8	4.8
Heulandite	3.0	2.7	3.3	3.0

### 2.2 Suction

After the disposal of HLW, the buffer material initially remains unsaturated by emanating decay heat generated by the spent fuel in the canister. However, over time, because of heat dissipation to the near-field rock and inflow of groundwater, the canister temperature decreases, thereby resaturating the buffer material. The degree of groundwater infiltration into the buffer material varies depending on the water potential (*ψ*), which is expressed as the energy level of water in the buffer material. As water moves from a high volume to a low volume region, the energy balance is attained. The total water potential (*ψ*_*t*_) can be calculated as follows [17]:

(1) $$\begin{array}{c} \psi_{t}=\psi_{p}+\psi_{o}+\psi_{m}+\psi_{g},\# \end{array}$$

where the pressure potential (*ψ*_*p*_) can be derived by applying an external pressure to the water such that the pressure potential equals zero when no external pressure is applied. The osmotic potential (*ψ*_*o*_) can be related to the solution concentration, which is proportional to the solute concentration such that the potential decreases during osmosis when the solute concentration increases. The matric potential (*ψ*_*m*_) is derived from the energy difference between the free water and that adsorbed by the buffer material particles, and the gravimetric potential (*ψ*_*g*_) is the energy required for a small amount of water to move against gravity. The *ψ*_*m*_ value of the saturated buffer material is close to zero, because the energy state of the water in the clay approaches that of free water. For compacted bentonite buffer material, *ψ*_*m*_ is much higher than *ψ*_*p*_, *ψ*_*o*_, and *ψ*_*g*_ and therefore can generally be used to indicate the overall water potential [17]. Suction (*s*) is represented as the negative of water potential:

(2) $$\begin{array}{c} s=-\psi_{t}\;.\# \end{array}$$

When measuring the water potential of the bentonite buffer material, considering the circumstances of engineered barriers, a capacitive thin-film polymer humidity sensor, which is effective under both high-temperature and pressure conditions, is preferred over thermocouple psychrometry [18].

### 2.3 Experimental apparatus for suction

**Fig. 1:**
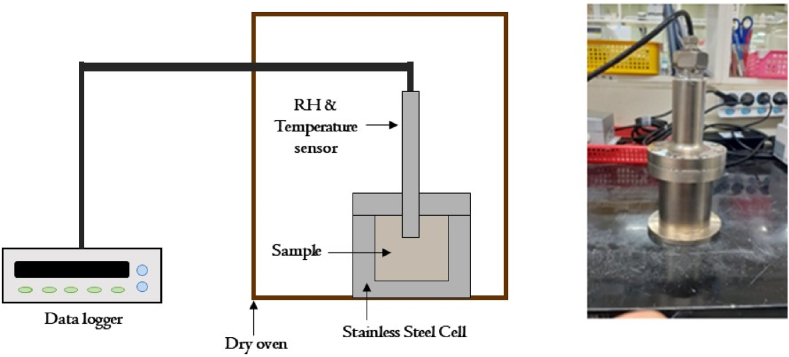
Experimental apparatus for suction measurement (unit (mm)).

The suction of KJ bentonite buffer material was measured using a vapor equilibrium (VE) method. This method assumes that the water potentials of the water and vapor in the pores of a sample are at equilibrium. The VE method is more suitable for measuring the suction of bentonites with high water-suction properties [11,14,18] than the axis translation method, which applies air pressure up to 1.5 MPa to a sample to increase the pressure gradually. The suction was calculated using the relative humidity (RH) through the Kelvin equation as follows:

(3) $$\begin{array}{c} s=-\frac{{RT}_{a}}{v_{\omega 0}\omega_{v}}\ln\left(\frac{RH}{100}\right),\# \end{array}$$

where *R* represents the gas constant (8.3143 J/mol·K); *T*_*a*_ and $\nu_{\omega 0}$ represent the absolute temperature and specific volume of water (0.001 m^3^/kg), respectively; and $\omega_{\nu }$ represents the molar mass of water (18.016 kg/kmol) [19]. The temperature and RH (VAISALA HMT 334) at the center of the sample were measured using sensors (Fig. 1). A silicon O-ring was installed between the cell body and cover to limit the leakage of evaporated moisture present inside the bentonite under high temperature conditions (≥100 °C). Before inserting the RH sensor into the sample, Teflon tape was attached to the cover of the container and sensor connection component to prevent water vapor leakage. The RH was measured at a specific temperature until it plateaued; thereafter, a constant RH value was applied to Eq. (3) to derive the suction of the buffer material. The RH values plateaued due to the inability of moisture to evaporate from the bentonite because the inner parts of the experimental system were well sealed.

### 2.4 Experimental apparatus for hydraulic conductivity

**Fig. 2:**
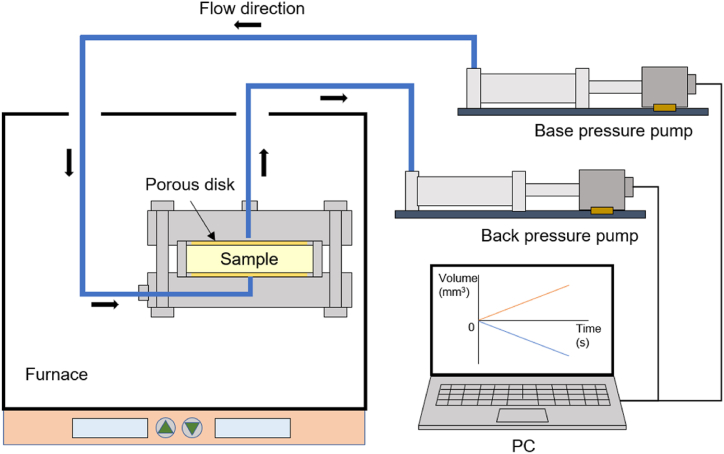
Experimental apparatus for hydraulic conductivity measurement.

Hydraulic conductivity, which significantly influences the suppression of nuclide outflow, is an important factor in designing a bentonite buffer material. To prevent nuclide leakage to the greatest extent possible, groundwater penetration from the near-field rock into the canister through the bentonite buffer material should be minimized, thereby ensuring that the groundwater bypasses the deposition hole without penetrating it. Additionally, nuclide advection through the groundwater must be prevented. Therefore, the hydraulic conductivity of bentonite buffer materials should be lower than that of the near-field rock. To measure the hydraulic conductivity of KJ bentonite buffer materials considering temperature increase, the experimental system shown in Fig. 2 was fabricated. The KJ bentonite buffer material with a thickness of 1 cm, diameter of 5 cm, and dry density of 1.6 g/cm^3^ was placed in a confinement cell with porous disks installed on the upper and lower surfaces. Two hydraulic pumps (base and back pressure pumps) were used to apply distilled water pressure to the bottom (1.2 MPa) and top (0.2 MPa) of the sample to generate a hydraulic head difference of 1 MPa. The hydraulic conductivity (*K*) of the sample was calculated using Darcy's law based on the flow rate (*Q*) into the back pressure pump as follows:

(4) $$\begin{array}{c} Q=K\frac{\Delta h}{L}A,\# \end{array}$$

where *Δh* is the hydraulic head difference, *L* is the thickness of the sample, and *A* is the cross-sectional area of the sample. The temperature of the sample was controlled by placing the cell containing the sample in a dry oven at 20 °C, 40 °C, 60 °C, 90 °C, 100 °C, 120 °C, 125 °C, and 150 °C.

## 3 Results and discussion

**Fig. 3:**
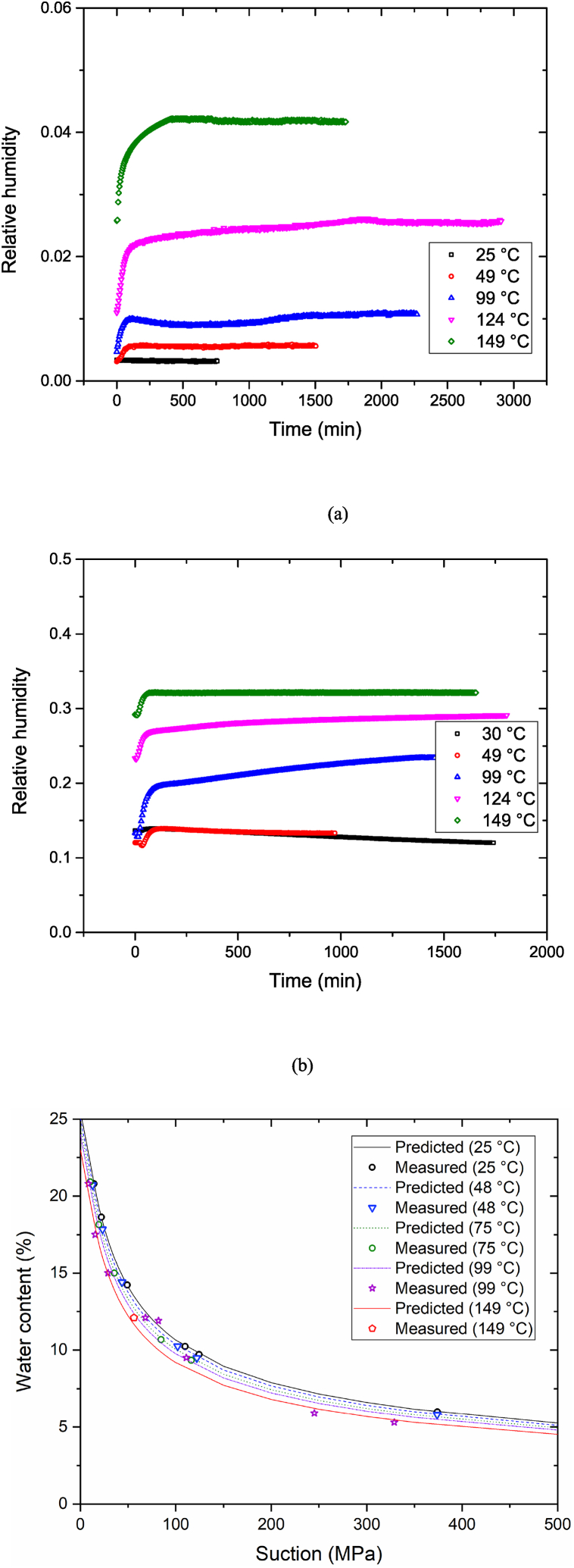
RH variation relative to increase in temperature. Initial water content: (a) w = 0, (b) w = 0.059, and (c) w = 0.121.

**Fig. 4:**
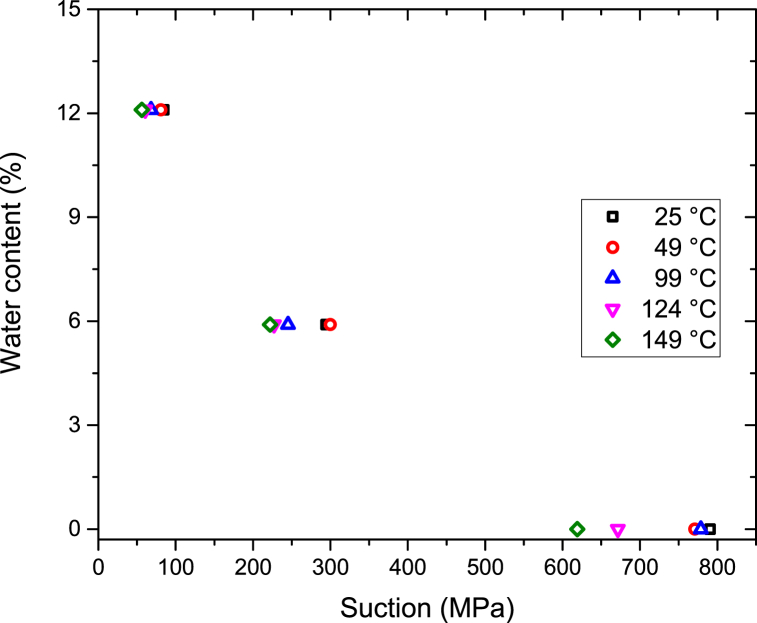
Suction variation relative to increase in temperature.

Kim et al. [15] reported that the water content of a buffer material was 0.05–0.06 when the temperature reached approximately 100 °C. The suction measurements over a temperature range (25 °C-149 °C) were performed using compacted bentonite samples with water content values of 0, 0.06, and 0.12, where the initial water content of newly produced bentonite was 0.12. The initial dry density was 1.61 (±1%) g/cm^3^. Fig. 3(a–c) shows the change in RH of the bentonite buffer material over time according to the three water content values. The RH of the sample increased as the temperature increased, because the water in the sample was vaporized in the cell. This phenomenon shows that water in the bentonite layers moves to the macropores of the bentonite aggregates, thereby increasing the saturation degree [17]. We observed that the suction decreased as temperature increased (Fig. 4). The lowest suction corresponded to the highest RH at 150 °C. In the sample with zero water content, the decreasing suction trend was steeper relative to the increase in temperature than that in the presence of water at temperatures >100 °C. In contrast, the sample with zero water content exhibited a marginal suction change at temperatures <100 °C due to the adsorbed water in the bentonite particles. Bentonite particles constitute one gibbsite between two silica sheets [20]. Free water can be drained through the capillary pores of bentonite; however, adsorbed water, which is the residual water concentrated by adsorptive forces [21], in the micropores of bentonite particles is difficult to drain. Generally, free water is removed from the capillary pores in soil upon drying in an oven at 110 °C [22]. Therefore, to remove the adsorbed water of bentonite, the bentonite must be dried at 110 °C-150 °C. Through this temperature range, suction decreases as adsorbed water moves to the capillary pores.

**Fig. 5:**
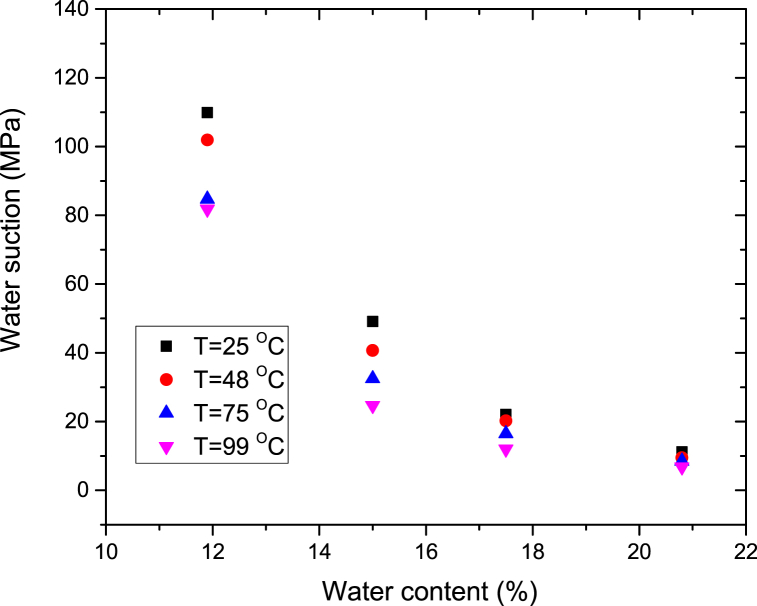
Experimental SWCC results obtained by Yoon et al. [14].

The experimentally derived change in suction relative to the temperature of the bentonite buffer material can be expressed using the modified van-Genuchten SWCC equation [23], shown in Eq. (5):

(5) $$\begin{array}{c} \omega =\left(an^{b}{e^{-c}}^{\left(T-T_{0}\right)}\right){\left[1+{\left(\frac{s}{P_{0}e^{-n\left(n-n_{0}\right)}e^{-\alpha \left(T-T_{0}\right)}}\right)}^{\frac{1}{1-\lambda }}\right]}^{-\lambda },\# \end{array}$$

where *ω*, *n*, *s*, and *T* represent the water content (%), porosity, suction (MPa), and temperature (°C), respectively. To utilize Eq. (5), the fitting coefficients were calculated using results generated during the current and a previous study [14]. Fig. 5 shows previous experimental SWCC results at 25 °C-99 °C with high initial water content that were obtained by Yoon et al. [14].

**Fig. 6:**
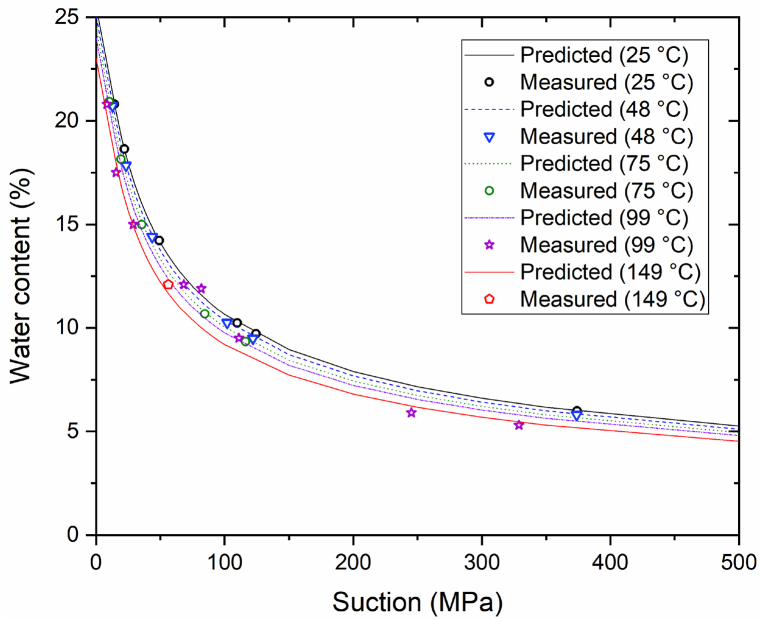
Comparison of experimental and predicted values.

The resulting values of *a*, *n*, *b*, *c*, *P*_*0*_, *λ*, *n*_*0*_, *α*, and *T*_*0*_ were 32.55, 0.406, 0.2692, 0.001, 14.96, 0.310, 0.406, 0.001, and 25 °C, respectively, where *a*, *b*, *c*, *P*_*0*_, *λ*, and *α* are the fitting parameters shown in Table 2. Fig. 6 compares the results obtained using the modified van-Genuchten SWCC equation (Eq. (5)) to the actual experiment results. The average relative error between the derived and experimental results was approximately 6%, which indicates that the modified equation is applicable at temperatures as high as 150 °C. Eq. (5) was fitted significantly in the temperature range 25 °C-149 °C, although the temperature range in Eq. (5) was 20 °C-80 °C.

**Table 2:** Parameter values for the modified van-Genuchten SWCC equation.

Parameter	Value
Fitting parameter, *a*	32.55
Porosity, *n*	0.406
Fitting parameter, *b*	0.2692
Fitting parameter, *c*	0.001
Fitting parameter, *P*_*0*_	14.96
Fitting parameter, *λ*	0.310
Initial porosity, *n*_*0*_	0.406
Fitting parameter, *α*	0.001
Initial temperature, *T*_*0*_ (°C)	25

**Fig. 7:**
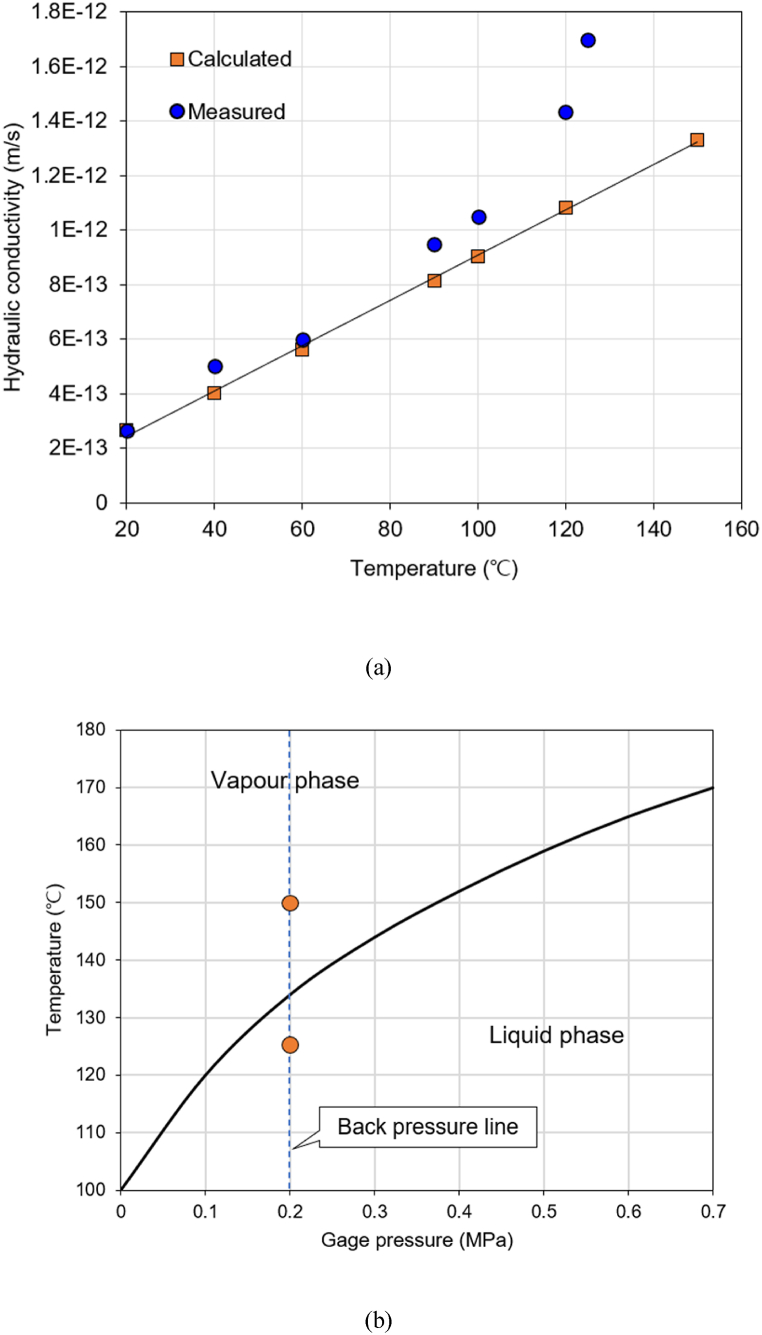
Hydraulic conductivity variation according to temperature increase. (a) Calculated and measured hydraulic conductivity. (b) Phase of water according to temperature and pressure [25].

The hydraulic conductivity of the KJ bentonite buffer material increased as the temperature increased (20 °C-150 °C). The hydraulic conductivity relative to the increasing temperature could also be calculated using Eq. (6), which considers the kinematic viscosity coefficient and changing water density relative to the temperature [9].

(6) $$K_{t}=K_{20{}^{\circ}C}\frac{V_{20{}^{\circ}C}}{v_{t}}\#$$

where *K*_*t*_ and *K*_*20*_ °C represent the hydraulic conductivities at a given temperature and at 20 °C, respectively, and *ν* denotes the kinematic viscosity (m^2^/s), which is the dynamic viscosity divided by density. The experimental and calculated results showed similar trends for temperatures ≤100 °C (Fig. 7(a)). Moreover, the experimentally obtained hydraulic conductivity slope at 30 °C-125 °C corresponded with the results reported by Daniels et al. [24]. However, this measured value was approximately 100 times larger than the calculated value around 150 °C, which can be attributed to the influence of the vaporized water. The pressure difference between the top and bottom of the sample was 1 MPa; however, the pressure at the top was 0.2 MPa, and the pressure gradient close to 0.2 MPa appeared from the bottom to the top of the sample. For water not to boil at 150 °C, this pressure should be > 0.4 MPa; therefore, the vaporization phenomenon probably occurred at the top of the sample (Fig. 7(b)) [25], and vapor flow could be dominated [26]. Accordingly, it can be inferred that this sharp increase occurred because of the considerably higher hydraulic conductivity of gas compared to that of liquid. Therefore, it is thought that there was an 30%–40% difference between measured and calculated values, especially at 100 °C-125 °C. Moreover, the increased difference between the measured and calculated hydraulic conductivities at 120 °C and 125 °C can be attributed to the varying intermolecular forces of the partially vaporized liquid water molecules because of the pressure gradient inside the sample. In terms of the structure of the sample, as the temperature increases, the volume expansion of the Ca-bentonite decreases because of dehydration [23]; therefore, the size of the pores increases with increasing temperature owing to the internal swelling of the sample under confined conditions. Consequently, the hydraulic conductivity of the sample increases nonlinearly as the temperature increases, not only because of molecular motion, but also because of the structural change inside the sample.

Subsequently, the hydraulic properties of KJ bentonite buffer materials with other bentonite types such as MX-80 (Na-type), which is commercially used as a buffer material globally, were compared. Daniel et al. [24] measured the hydraulic conductivity of MX-80 bentonite at 20 °C-200 °C and found it to be 10 times smaller than that of KJ bentonite. Park et al. [13] explained that the increase in pore size owing to the low swelling effect of Ca-type bentonite can improve the hydraulic conductivity. Considering suction, Villar and Gòmez–Espina [27] measured the suction of MX-80 bentonites with different water contents at 20 °C-120 °C. At 0.12–0.13 water content, the suction of KJ bentonite buffer material was 20%–30% larger than that of MX-80 bentonite. KJ bentonite is also known to exhibit higher relative hydraulic conductivity than MX-80 [28]. The suction and hydraulic conductivity of the KJ bentonite buffer material showed only a marginal correlation in comparison with those of the MX-80 bentonite. Although the KJ bentonite had a hydraulic conductivity approximately 10 times greater and montrillonite content 30% lower than those of MX-80, it can be used as a buffer material, because the hydraulic conductivity is sufficiently smaller than that of general rock-mass. Moreover, the suction was 20–30 times greater than that of MX-80, which is advantageous for taking groundwater in a deep geological environment.

## 4 Conclusion

This study aimed to investigate the changes in the suction and hydraulic conductivity of KJ bentonite buffer material at high temperatures (>100 °C). The bentonite samples were analyzed with initial saturation of 0, 0.23, and 0.47 and a dry density of 1.61(±1%) g/cm^3^ in accordance with previously published results obtained at temperatures <100 °C. A measurement system was established to evaluate the suction and hydraulic conductivity of the bentonite buffer material at 20 °C-150 °C.

As the temperature increased, the RH of the bentonite buffer material increased, whereas the suction decreased. This finding can be attributed to the evaporation of the moisture within the bentonite samples with increasing temperature, thereby increasing the amount of water vapor in the air. Although the dry sample exhibited no change in suction at 20 °C-100 °C, the suction decreased by approximately 20% in the range 100 °C-150 °C. This result may be attributed to the movement of adsorbed water within the bentonite to the capillary pores at temperatures >100 °C. Additionally, the suction of samples with initial saturation of 0.23 and 0.47 decreased by approximately 10%–20% in the temperature range of 100 °C-150 °C. The suction measurements experimentally obtained during this study (>100 °C) and those obtained in a previous study (<100 °C) were substituted into a modified van-Genuchten SWCC equation, which considers temperature to calculate the fitting parameters. The relative error between the experimental and model values was approximately 6%, which indicates that the modified equation is applicable at temperatures as high as 150 °C. The suction of the KJ bentonite buffer material was 20%–30% greater than that of MX-80 bentonite. Furthermore, the hydraulic conductivity of the KJ bentonite buffer material when measured at 20 °C-150 °C increased as the temperature increased. Although the experimental results were approximately consistent with the model results up to 100 °C, they were significantly greater than the model results at higher temperatures: at 150 °C, they were 100 times greater than the model results. Considering that the upper pressure applied inside the cell should be 0.4 MPa to prevent water from boiling at 150 °C, the lower 0.2 MPa upper pressure likely caused vaporization at the top of the sample, thereby increasing the hydraulic conductivity significantly with increasing pore size due to dehydration caused by the increase in temperature. In actual deep disposal environments at depths of approximately 500–1000 m, the hydrostatic pressure will be significantly greater as it is proportional to depth (5–10 MPa). Accordingly, the hydraulic conductivity of the buffer material is not expected to increase because of vaporization at 150 °C. The hydraulic conductivity of the KJ bentonite buffer material was on the order of 10^−13^ m/s, which is acceptable for a buffer material.

The results obtained here will be fundamental in facilitating the safety evaluation of repositories in accordance with future temperature increases to design effective buffer materials. Complex properties such as the thermal and mechanical characteristics of bentonite buffer materials must also be investigated beyond 100 °C to evaluate their adaptability as buffer materials.

## Author contribution statement

Seok Yoon: Conceived and designed the experiments; Performed the experiments; Analyzed and interpreted the data; Contributed reagents, materials, analysis tools or data; Wrote the paper. Jun-Seo Jeon: Analyzed and interpreted the data. Gi-Jun Lee: Performed the experiments; Analyzed and interpreted the data; Contributed reagents, materials, analysis tools or data; Wrote the paper.

## Data availability statement

Data included in article/supp. material/referenced in article.

## Additional information

No additional information is available for this paper.

## Declaration of competing interest

The authors declare that they have no known competing financial interests or personal relationships that could have appeared to influence the work reported in this paper.

## References

[1] Simmons G.R., Baumgartner P. (1994).

[2] Wilson J., Bond A. (2016).

[3] Kim J.S., Cho W.J., Park S., Kim G.Y., Baik M.H. (2019). A review on the design requirement of temperature in high-level nuclear waste disposal system: based on bentonite buffer. J. Korean Tunn. Undergr. Sp. Assoc..

[4] SKB (2009).

[5] Wersin P., Johnson L.H., McKinley I.G. (2007). Performance of the bentonite barrier at temperatures beyond 100 °C: a critical review. Phys. Chem. Earth.

[6] Prikryl R., Weishauptová Z. (2010). Hierarchical porosity of bentonite-based buffer and its modification due to increased temperature and hydration. Appl. Clay Sci..

[7] Park T.J., Seoung D. (2021). Thermal behavior of groundwater-saturated Korean buffer under the elevated temperature conditions: in-situ syncrotron X-ray powder diffraction study for the montmorillonite in Korean bentonite. Nucl. Eng. Technol..

[8] Cho W.J., Kim G.Y. (2016). Reconsideration of thermal criteria for Korean spent fuel repository. Ann. Nucl. Energy.

[9] Das B.M. (2006).

[10] Cho W.J. (2017).

[11] Jacinto A.C., Villar M.V., Gómez-Espina R., Ledesma A. (2009). Adaptation of the van Genuchten expression to the effect of temperature and density for compacted bentonites. Appl. Clay Sci..

[12] Wan M., Ye W.M., Chen Y.G., Cui Y.J., Wang J. (2015). Influence of temperature on the water retention properties of compacted GMZ01 bentonite. Environ. Earth Sci..

[13] Park S., Yoon S., Kwon S., Lee M.S., Kim G.Y. (2021). Temperature on the thermal and hydraulic conductivity of Korean bentonite buffer material. Prog. Nucl. Energy.

[14] Yoon S., Jeon J.S., Go G.H., Kim G.Y. (2020). An evaluation of soil-water characteristic curve model for compacted bentonite considering temperature variation. J. Korean Geotech. Soc..

[15] Kim K.I., Lee C., Kim J.S., Cho D. (2021). A numerical analysis to estimate disposal spacing and rock mass condition for high efficiency repository based on temperature criteria of bentonite buffer. J. Korean Tunn. Undergr. Sp. Assoc..

[16] Lee C., Yoon S., Cho W.J., Jo Y., Lee S., Jeon S., Kim G.Y. (2019). Characterization of KURT granite and Gyeongju bentonite. J. Nucl. Fuel Cycle Waste Technol..

[17] Cho W.J. (2019).

[18] Lee J.O., Kim G.Y., Yoon S. (2017).

[19] Nguyen-Tuan L. (2014).

[20] JNC (1999).

[21] Revil A., Lu N. (2013). Unified water isotherms for clayey porous materials. Water Resour. Res..

[22] Lee C. (2021).

[23] Villar M.V., Gómez-Espina R., Lloret A. (2010). Experimental investigation into temperature effect on hydro-mechanical behaviours of bentonite. J. Rock Mech. Geotech. Eng..

[24] Daniels K.A., Harrington J.F., Zihms S.G., Wiseall A.C. (2017). Bentonite permeability at elevated temperature. Geosciences.

[25] Sonntag D., Heinze D. (1982).

[26] Zhai Q., Ye W., Rahardjo H., Satyanaga A., Dai G. (2021). Theoretical method for the estimation of vapour conductivity for unsaturated soil. Eng. Geol..

[27] Villar M.V., Gómez-Espina R., Schanz T. (2007). Experimental Unsaturated Soil Mechanics.

[28] Yoon S., Kim M.S., Kim G.Y., Lee S.R. (2021). Contemplation of relative hydraulic conductivity for compacted bentonite in a high-level radioactive waste repository. Ann. Nucl. Energy.

